# A South African Breast Implant-Associated Anaplastic Large Cell Lymphoma: Clinical Presentation and Six-Year Follow-Up

**DOI:** 10.1155/2022/4162832

**Published:** 2022-05-31

**Authors:** Alexandra Grubnik, Yastira Ramdas, Barend Van der Bergh, Simon Nayler, Carol-Ann Benn, Bernardo L. Rapoport

**Affiliations:** ^1^Netcare Breast Care Centre of Excellence, Milpark Hospital, 9 Guild Road Parktown, South Africa; ^2^Department of Immunology, Faculty of Health Sciences, University of Pretoria, Corner Doctor Savage Road and Bophelo Road, Pretoria 0002, South Africa; ^3^Drs Gritzman & Thatcher Inc. Laboratories, 4 Main Street, Bordeaux, Randburg, Johannesburg 2194, South Africa; ^4^University of the Witwatersrand, Donald Gordon Medical Centre, 21 Eton Road, Parktown Johannesburg, 2193, South Africa; ^5^Helen Joseph Hospital Breast Care Clinic, Department of Surgery, Faculty of Health Sciences, University of the Witwatersrand, 1 Perth Road, Auckland Park, Johannesburg, 2092 Gauteng, South Africa; ^6^The Medical Oncology Centre of Rosebank, 129 Oxford Road, Johannesburg 2196, South Africa

## Abstract

Breast augmentation is the most common surgical procedure for women globally, with 1,795,551 cases performed in 2019. Breast implant-associated anaplastic large cell lymphoma (BIA-ALCL) is highly uncommon, with 733 reported cases as of January 2020. In South Africa, there are less than 4000 breast augmentation surgeries annually. This case presents the first case report documentation of a South African woman diagnosed with BIA-ALCL. The patient was a 61-year-old woman who consulted the Breast Care Centre of Excellence in Johannesburg in 2015. She had a prior history of bilateral augmentation mammoplasty with subsequent implant exchange. The patient presented with periprosthetic fluid with a mass-like enhancement on the left breast. Aspiration of the mass-like fluid was positive for CD45, CD30, and CD68 and negative for CD20 and ALK-1, indicative of BIA-ALCL. Surgical treatment included bilateral explantation, complete capsulectomies, and bilateral mastopexy. Macroscopic examination of the left breast capsulectomy demonstrated fibrous connective tissue. The histological examination of the tumor showed extensive areas of broad coagulative necrosis with foamy histiocytes. Immunohistochemistry examination of this tumor showed CD3-, CD20-, and ALK-1-negative and CD30- and CD68-positive stains. PCR analysis for T-cell clonality showed monoclonal T-cell expansion. These findings confirm the presence of BIA-ALCL. The patient recovered well after surgery and did not require adjuvant therapy. A patient with a confirmed diagnosis of BIA-ALCL was successfully treated with explantation and complete capsulectomy. She was followed up regularly for six years, and the patient remains well and in remission.

## 1. Background

Breast augmentation remains the most common procedure globally, with 1,795,551 performed in 2019 [1]. An estimated 10 million women have implants globally [2]. In South Africa, 3297 breast augmentation cases with silicone implants were recorded in 2016 [2]. Breast implant-associated anaplastic large cell lymphoma (BIA-ALCL), a subtype of T-cell non-Hodgkin's lymphoma, is associated with textured surface breast implants [3, 4]. The cause of BIA-ALCL remains unknown, although several theories, including biofilm, viral, shedding of particles, and genetic predisposition, have been proposed [5–9].

BIA-ALCL is a novel malignant entity. It was first described in 1997 and reported by the Food and Drug Administration (FDA) in 2011 [10--11]. In 2016, the World Health Organization (WHO) categorized it as a unique disease [10]. NCCN diagnosis and treatment guidelines were published in 2016 and updated in 2019 [11].

As of January 2020, 733 cases of BIA-ALCL were reported to the FDA, including 36 deaths [10]. The incidence and risk of BIA-ALCL have risen considerably from early reports of 1 per million to current estimates of between 1/2,832 and 1/30000, and in specific cohorts, an incidence of as high as 1/355 was reported [10, 12, 13]. The disease incidence mainly depends on the “population” (implant type and characteristics) examined and increased awareness of the disease [14]. To our knowledge, this is the first peer-reviewed case report of a patient with BIA-ALCL in South Africa and the African continent.

## 2. Case Presentation

**Figure 1:**
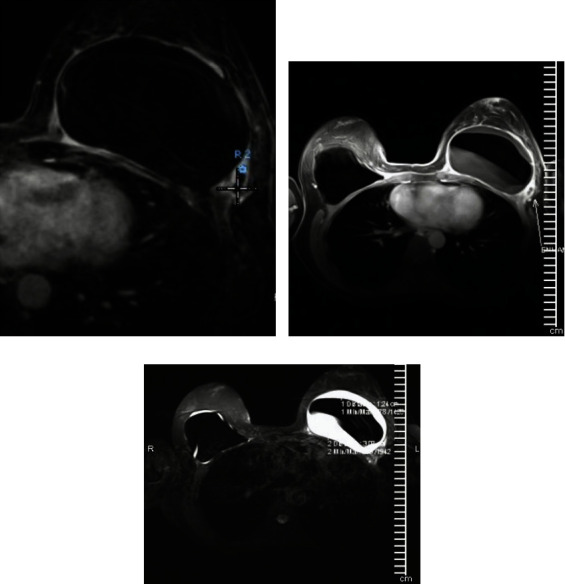
(a–c) MRI images of periprosthetic fluid and mass.

**Figure 2:**
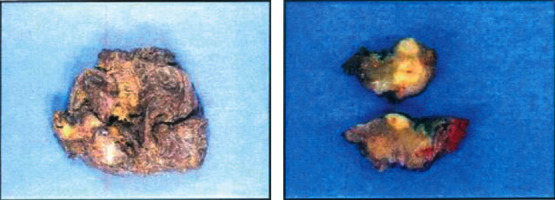
Macroscopy examination: (a) right breast capsulectomy with a mass of 47 g and (b) left breast capsulectomy and implant 294 g.

A 61-year-old woman initially had a bilateral breast augmentation with Nagor-textured silicone implants in 1994. She had bilateral implant exchange with Allergan, smooth implants in subpectoral pockets, and mastopexy in 2007. She presented to the Breast Care Centre of Excellence in Johannesburg in 2015 with initial swelling of the left breast. Mammography, ultrasound, and MRI revealed a periprosthetic fluid collection and mass-like enhancement on the left breast at the 4 o'clock region showing sustained peripheral enhancement (Figure 1). Family history: the father died of colon cancer. Fluid was aspirated, and cytologic smears on a tissue block were analyzed by hematoxylin-eosin stain and immunohistochemistry. The tumor cells were positive for CD45, CD3, and CD30. Additionally, CD68 was positive in a population of reactive background histiocytes. The tumor was negative for ALK-1, and CD20 was indicative of BIA-ALCL. Preoperative positron emission topography (PET) demonstrated level 1 lymph nodes on the left. The patient was discussed at a multidisciplinary team (MDT) meeting at the Breast Care Centre of Excellence. The patient underwent “en bloc” capsulectomy, which includes removing the implant, capsule, and eventual mass surrounded by a margin of healthy tissue. Surgery and recovery were uneventful. Macroscopic examination of the right breast capsulectomy tissue confirmed a fibrotic capsule showing typical pseudosynovial metaplasia. The left breast capsulectomy revealed similar fibrous connective tissue with the tumor biopsy showing extensive areas of broad coagulative necrosis with foamy histiocytes (Figure 2).

**Figure 3:**
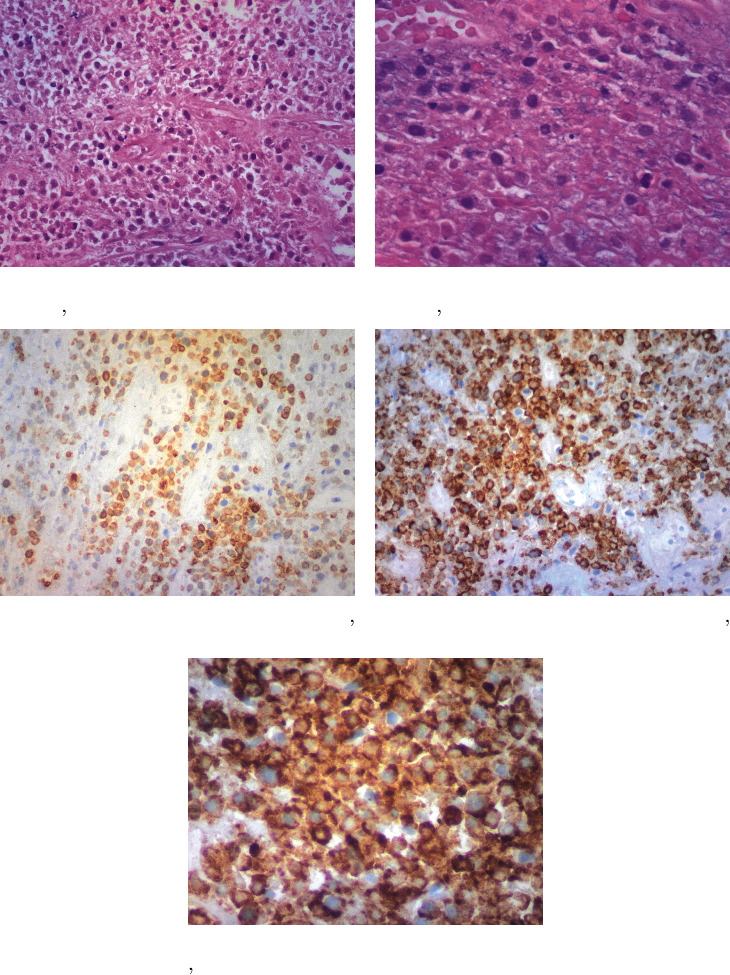
(a–e) Microscopic examination.

**Figure 4:**
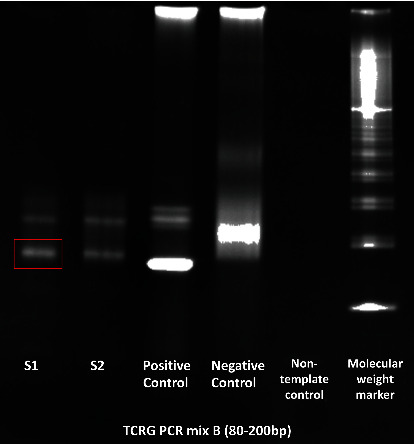
Gel electrophoresis showing clonal band for T-cell receptor gamma. The tumor is shown in the S1 band.

The tumor infiltrated the superficial aspect of the capsule only. The ink margins were clear. Immunohistochemistry examination of this tumor showed CD3-, CD20-, and ALK-1-negative and CD3-, CD30-, and CD68-positive stains. PCR analysis for T-cell clonality showed monoclonal T-cell expansion (Figures 3(a)–3(e)) [15]. DNA was extracted from the submitted sample using QIAGEN® QIA amp DNA FFPE tissue kit. Clonality was performed by the IdentiClone™ TCRB+TCRG T-clonality Assay. The PCR product was electrophoresed with the Bio Rad® system. The PCR showed monoclonal bands (Figure 4). The following abnormalities were detected: **A**-V*β*+J*β*1+J*β*2 regions TCRB; **B**-VB+J*β*2 regions TCRB; **C**-D*β*+J*β*1+J*β*2 regions TCRB; **A**-V*δ*1-8, V*δ*10, + multiple J*δ* regions TCRG; and **B**-V*δ*9, V*δ*11+multiple J*δ* regions TCRG.

These abnormalities were detected on the gel electrophoresis according to the Euroclonality/Biomed-2 guidelines for interpretation and reporting Ig/TCR clonality in suspected lymphoproliferation [16].

Histological examination of a gland removed below the low age of the pectoralis minor muscle (level 1 axillary lymph node) showed a reactive lymph node without evidence of lymphoma involvement.

Following surgery, the patient noted reduced swelling and pain. A six-week follow-up examination revealed no inflammation, reduced pain levels, and no evidence of recurrence. No axillary or supraclavicular pathological lymphadenopathy was seen.

Follow-up visits consisted of physical examination, full blood counts, serum chemistry tests, ultrasound of the breast, chest roentgenograms, and ultrasound of the abdomen and pelvis or computerized body tomography. The patient was followed up at a six-month interval for five years. Currently, the patient is followed up yearly, and she remains in remission at year six.

## 3. Discussion

BIA-ALCL was first reported in 1997 [17]. The risk of BIA-ALCL increases yearly, with breast augmentation being the most common surgery performed annually for women. Still, there are global discrepancies between surgeon preferences, costs, and various implant technologies [14].

The reported incidence of BIA-ALCL is increasing. This may be due to increased disease awareness. BIA-ALCL is exclusively associated with textured implants, with no confirmed cases in patients with only smooth devices to date [11, 15, 18, 19]. Like our patient, patients with smooth implants at the time of BIA-ALCL diagnosis were found to have a mixed implant history with the previous textured devices. In 2019, the FDA recommended a global voluntary recall of Allergan Biocell textured implants, following findings of increased BIA-ALCL risk with these devices [15]. Shortly following this announcement, several regulatory boards, including France and Canada, have banned macrotextured implants, thus significantly increasing public awareness of BIA-ALCL. Another possible reason is underestimating the actual incidence in published studies with a short median follow of 2-4 years. BIA-ALCL usually occurs after a median of 6-13 years [11, 13].

Breast non-Hodgkin's lymphoma (NHL) is primarily B-cell in origin (95%). T-cell lymphomas of the breast have 3 different subtypes, including anaplastic lymphoma kinase- (ALK-) negative ALCL, ALK-positive ALCL, and cutaneous ALCL [20].

Epidemiologically, non-Hodgkin's lymphomas associated with the breast account for less than 2% of extranodal NHL and less than 1% of NHL, with most presenting as diffuse B-cell, marginal zone lymphoma and follicular lymphoma [21]. BIA-ALCL is a rare subtype of T-cell lymphoma pathologically associated with CD30-positive, ALK-negative ALCL. The risk of women with implants contracting BIA-ALCL is relatively low, and it was only recently classified as a unique disease [22]. A recent European study by the Committee on Device Safety and Development (CDSD) reported 420 cases in Europe, with an overall prevalence of 1 : 13,745 cases in the 28 member states of the European Union (EU-28). Countries where specific measures have been implemented to tackle BIA-ALCL account for 61% of the EU-28 population and actively reported 382 cases with an overall prevalence of 1 : 9121 [23]. The disease is treated primarily surgically and is associated with a good prognosis with an overall survival rate of 93-94% at three years and 89-91% at five years [24–26].

The pathogenesis of BIA-ALCL remains unknown; however, chronic inflammation and abnormal immune response may be associated with this disease. The cause for such a response may be bacterial biofilm. Hu et al. found Ralstonia pickettii in more than half of diseased capsules [5]. Kadin et al. demonstrated that BIA-ALCL cells might be derived from lymphocytes with Th1/Th17 polarization in capsular tissues and surrounding seromas, suggesting a chronic bacterial antigen stimulation of the liposaccharide coat of Gram-negative bacteria and a persistent T-cell proliferation might support BIA-ALCL initiation and disease progression [27]. Another theory suggests that the immune response is caused by silicone particles shed from the surface of textured implants [8]. In a separate report, Kadin et al. showed that the BIA-ALCL microenvironment is characterized by high levels of interleukin-13 and IgE, suggesting that the immune response underlying BIA-ALCL has the profile of chronic allergic reaction [28]. Di Napoli et al. reported novel insights on the pathogenesis of BIA-ALCL [7]. By performing gene expression profiling, the investigators compared the transcriptional profiles of BIA-ALCL with those of normal T-cells and other peripheral T-cell lymphomas. Compared to normal CD4+ T-cells, BIA-ALCL was associated with an upregulation of genes involved in cell motility processes, including chemokine receptor 6 (CCR6), MET, hepatocyte growth factor (HGF), and chemokine (C-X-C motif) ligand 14 (CXCL14) [7]. Several reports suggest activation of the JAK-STAT3 pathway in 13-26% of BIA-ALCL cases [9].

BIA-ALCL typically presents with late seroma in 60-90% of cases [16]. It may also present with a solid mass in the capsule adjacent to the breast implant in 8-24% of cases and, less commonly, with lymphadenopathy (4-12%) or local and systemic symptoms like skin rash and fevers (<5%) [11, 21, 26]. The majority of cases present at an average of 8-10 years after implantation [29]. Patients with suspected BIA-ALCL typically present with unilateral breast swelling. An ultrasound should be performed, and if fluid is present, fine needle aspiration of a minimum of 50 ml of fluid should be undertaken [11]. Fluid is sent for cytology, CD30 immunochemistry, and flow cytometry [11, 29]. A core needle biopsy is recommended if a solid mass is present [11]. A positron emission tomography scan (PET CT scan) is performed for staging before surgery to avoid distortion for 2-3 months afterward [11].

Once the diagnosis is made, the patient should be managed by a multidisciplinary team comprising oncologists, pathologists, radiation oncologists, surgeons, and plastic surgeons. Staging investigations include serum chemistry, serum lactate dehydrogenase, C-reactive protein, *β*2 microglobulin, bone marrow examination (aspiration, biopsy, and flow cytometry), and PET CT scan [11]. However, there are limitations to the current diagnosis methods mentioned owing to patients that underwent aspiration of the effusion or patients undergoing implant and capsule removal, which can affect the CD30 immunochemistry [30]. A recent report by Di Napoli et al. outlines biomarkers that might help to differentiate between BIA-ALCL from all types of benign late seromas. It has been reported that benign effusions may also have an oligo-/monoclonal expansion of CD30+ cells, challenging the diagnosis of BIA-ALCL [31]. Di Napoli et al. applied a multiplexed immuno-based assay to BI-ALCL seromas. The researchers found that BI-ALCL is characterized by a Th2-type cytokine milieu associated with significantly high levels of IL-10, IL-13, and eotaxin, which discriminate BIA-ALCL from all types of reactive seroma. Additionally, the authors found that a cutoff value of IL10/IL-6 ratio of 0.104 is associated with a specificity of 100% and a sensitivity of 83% in distinguishing effusions due to BIA-ALCL [31].

Treatment guidelines for the management of BIA-ALCL are well established [11]. There is a consensus reached between the surgical and oncology communities. In most cases, complete surgical excision of the lymphomatous tumor mass and the breast implant with en block capsulectomy is recommended. Complete surgical excision prolongs overall survival and event-free survival [26]. Additionally, if BIA-ALCL with positive lymph nodes is present, adjuvant chemotherapy may be required, with brentuximab vedontin considered the preferred first line [32]. If residual disease is present, radiotherapy is advised [11, 33]. The prognosis is favorable; a study of 87 diagnosed BIA-ALCL patients reported an overall survival of 94% and 91% at three and five years, respectively, with event-free survival recorded at 49% at five years [26]. Similar results are noted in smaller study groups, and a longer-term follow-up of 60 patients reported a median OS of 12 years, OS at three and five years measured at 97% and 92%, respectively [34].

Of interest is the fact that this manuscript is the first peer-reviewed documented case of BIA-ALCL reported in South Africa, confirmed after an extensive PubMed and literature review of cases prior to 2021.

## 4. Conclusion

This case describes the successful surgical treatment and six-year long-term follow-up of a BIA-ALCL South African patient.

## Data Availability

The data of this case report is available from the patient's medical records.
